# Are fluctuations in physical performance affected by contextual factors in women’s handball matches? An analysis using five-minute fixed phases

**DOI:** 10.5114/biolsport.2025.139086

**Published:** 2024-06-04

**Authors:** Carlos García-Sánchez, Rafael Manuel Navarro, Raúl Nieto-Acevedo, Alfonso de la Rubia

**Affiliations:** 1 Deporte y Entrenamiento Research Group, Departamento de Deportes, Facultad de Ciencias de la Actividad Física y del Deporte (INEF), Universidad Politécnica de Madrid, C/Martín Fierro 7, 28040 Madrid, Spain; 2 Faculty of Sports Sciences, European University of Madrid; 28670 Villaviciosa de Odón, Spain

**Keywords:** Monitoring, Fatigue, Wearable Device, External Load, Local Positioning System, Player Tracking

## Abstract

The purpose of this study was twofold: to analyse physical performance fluctuations throughout match play in women’s handball; and to investigate whether physical performance fluctuations are affected by contextual factors (i.e., level of the opponent and playing positions). Twenty-two female players from the Spanish 2^nd^ Division were monitored across 13 matches. Each match was divided into 5 min fixed phases. Total distance (TD), high-speed running (HSR) and PlayerLoad (PL) were collected using a local positioning system. The highest values of TD, HSR and PL were registered during the first 5 min phase of the match (p < 0.05, moderate-large effects), while the lowest values of TD and PL were registered in the last phase of the first half and for HSR in the last phase of the match (p < 0.001, large effects). Regarding level of the opponent, low-level teams elicited higher TD in the first 10 min of the match (p < 0.05, moderate effects). Conversely, matches involving high-level teams registered more TD and PL in the last phase of the match (p < 0.05, moderate effects). In relation to playing positions, wings showed the highest physical performance in all 5 min phases of the match, whereas the pivots showed the lowest physical performance. In the present study the physical performance decreased throughout the match and the fluctuations were strongly affected by the level of the opponent and playing positions. Therefore, handball coaches should incorporate strategies to mitigate fatigue within and between halves.

## INTRODUCTION

Handball is considered an intermittent team sport characterized by periods of high-intensity actions that are interspersed with lower intensity activities [1]. During a match, players must perform different general movements (e.g., walking, running, sprinting, jumping and changing of direction) and handball-specific actions (e.g., passing, catching, throwing and blocking) with frequent and strenuous body contact against the opponents [2, 3]. Therefore, analysing the physical performance of the players during official matches provides many advantages for coaches and practitioners to: (1), design short- and long-term training programmes to maximize performance, reduce injury risk and minimize the risk of overtraining (2) adapt and periodize weekly training loads to manage stress and recovery, (3) design physical training interventions during the microcycle considering the player role in the match (starter vs. non-starter), and (4) develop and implement individualized physical training programmes for each playing position [1, 4]. To accomplish this purpose, technical staff and sports scientists can use new monitoring tools with a good level of validity [5, 6] and reliability [7], such as a local positioning system (LPS) with ultra-wideband (UWB) technology or inertial measurement units (IMUs) (e.g., accelerometer, magnetometer and gyroscope) to measure and analyse different external load variables in real time.

The physical demands in handball have been quantified across male and female competitions such as ASOBAL Spanish League [8], LIQUI-MOLY Handball-Bundesliga [9], and Spanish Women’s 2^nd^ Division [10] in addition to the European Champions League Final Four [11] and men’s [12, 13] and women’s [14, 15] international tournaments. To summarize this information, a recent systematic review [4] indicated that elite handball players usually cover between 2000 and 4500 m per match, with high-intensity running and sprinting accounting for 5% to 15% of this distance [16–19]. Nevertheless, the external loads experienced by handball players are highly variable due to the influence of gender [4, 10], competition level [4, 11–15] and contextual factors such as playing position [8–15] or match halves [16–20].

In the last years, the traditional approach to analyse the physical demands in handball has been based on quantifying the average values of different external load variables (e.g., total distance covered, high-speed running, accelerations and decelerations) during the complete match [8–11]. However, this approach does not provide accurate information about the physical demands of smaller phases or periods of the matches [12, 21]. As a solution to the problem, different researchers have analysed the differences between the first and the second half of the matches [16–19]. In this regard, as in other team sports such as basketball [22], previous research has revealed that the physical performance decreases throughout a handball match [12, 14–19]. More specifically, some studies have demonstrated that the time spent in high-intensity running and in very-high metabolic power decreased from the first to second half [16–20]. Similarly, initial values of Player-Load/min declined throughout the halves [15]. Moreover, the total distance covered in the first ten minutes was slightly greater than that covered in the last ten minutes of the match [12]. Thus, according to previous research [14–20], this decline in physical performance throughout the match suggests that handball players probably experience fatigue during the match, as has been previously described in other team sports such as basketball [22] and soccer [23–25].

However, to the best of our knowledge, there is limited research that provides information about the fluctuations in physical performance during small phases or periods of women’s handball matches. Therefore, the aims of the present study were: (1) to analyse physical performance fluctuations throughout match play in women’s handball, and (2) to investigate whether physical performance fluctuations are affected by contextual factors (i.e., level of the opponent and playing positions).

## MATERIALS AND METHODS

### Design

We conducted a retrospective observational design study to analyse physical performance fluctuations registered during 5 min fixed phases in official women’s handball matches. The LPS data collected correspond to the average values of 5 min fixed phases registered during 13 official home matches from the Spanish 2^nd^ Division in the 2021–2022 season.

### Participants

Twenty-two female handball players from the same team participated in this study. Table 1 shows the anthropometric characteristics of the players for each playing position. During the season, players typically completed four or five handball training sessions, two or three strength training sessions and one match per week. All players were informed of the study requirements and provided written informed consent prior to the start of the study. Additionally, all the ethical procedures used in this study were in accordance with the Declaration of Helsinki and were approved by the Ethics Committee of the European University of Madrid (CIPI/18/195).

**TABLE 1:** Anthropometric characteristics of the players.

Playing Positions	n	Age (years)	Height (cm)	Body Mass (kg)	BMI (kg/m^2^)
Backs	14	20.9 ± 3.6	168.7 ± 3.9	65.4 ± 6.8	27.1
Pivots	4	21.0 ± 1.8	171.3 ± 4.8	79.1 ± 11.0	23.2
Wings	4	18.8 ± 0.5	162.0 ± 3.8	55.5 ± 4.3	21.1
All players	22	20.5 ± 3.1	168.0 ± 4.8	66.1 ± 10.1	23.4

### Procedures and data analysis

The LPS system (WIMU PRO, RealTrack System SL, Almería, Spain) was installed on the official handball court where the team played their home matches according to the user manual and previous studies [8, 10]. All players were already familiarized with the data-collection procedures during previous training sessions and friendly matches. Each player was fitted with a device on his back with an adjustable vest. The manufacturer’s specific software (SPRO, version 958, RealTrack System SL, Almería, Spain) was used to calculate the perimeter of the court to determine the effective playing time. Consistent with previous studies [8, 10, 21], playing time was recorded only when the players were inside the court, omitting periods when the match was interrupted. Additionally, only players completing a minimum of 60% of each fixed phase (^3^3 min for each 5 min time window) were included in the analysis [15, 21]. Due to stop-pages during match play, the duration of the match halves could be longer than 30 minutes [15, 19]. After the match, the LPS files were exported to a USB memory device and analysed using the manufacturer’s specific software. Finally, raw data were exported post-match in Excel format and imported into the statistical software for statistical analysis. At the end of this process, a total of 1166 individual LPS registers from 13 official home matches were collected.

### Contextual factors and external load variables

Two contextual factors were considered: (1) level of the opponent. Considering the final ranking of each team in the competition we established three tiers: ‘high-level teams’ (HLT) (1^st^ to 5^th^ place), ‘middle-level teams’ (MLT) (6^th^ to 10^th^ place), and ‘low-level teams’ (LLT) (11^th^ to 14^th^ place). These categories are similar to those reported previously [9, 26–28]; (2) playing positions: backs, pivots and wings. The number of matches and individual LPS registers for each contextual factor and playing position are shown in Table 2.

**TABLE 2:** Number of matches and individual LPS registers according to contextual factors.

Contextual factor	Variable	Matches	LPS registers
Level of the opponent	High-level teams	4	362
	Middle-level teams	5	453
	Low-level teams	4	351

Playing positions	Backs	13	650
	Pivots	13	153
	Wings	13	363

Three external load variables were collected: (1) total distance (TD) considering total distance covered by the player and expressed in metres; (2) high-speed running (HSR) corresponding to the distance covered above 18.1 km/h and expressed in metres; (3) PlayerLoad (PL) was expressed in arbitrary units (a.u.) and calculated as the square root of the sum of the squared instantaneous rates of change in acceleration in each one of the three planes divided by 100.

### Statistical analysis

Data in the text and figures are presented as means and standard deviations (M ± SD). Before carrying out the analyses, the Kolmogorov-Smirnov test was performed to confirm data distribution normality. Variance and sphericity assumptions were checked with the Levene and the Mauchly tests. In relation to the first aim of the study, a repeated-measures analysis of variance (ANOVA) with the Tukey post hoc test was used to examine the physical performance fluctuations (i.e., TD, HSR and PL) between different 5 min fixed phases of the match. Considering the second aim of the study, a two-way ANOVA with the Tukey post hoc test was used to evaluate the interaction between match phases and contextual variables (i.e., level of the opponent and playing positions) on the external load experienced by handball players. Furthermore, partial eta-squared (*ηp*^2^) was calculated for group effects with the following interpretation: > 0.01 *small*, > 0.06 *moderate*, and > 0.14 *large* [29]. Cohen’s effect sizes (ES) were calculated and interpreted using Hopkins’ categorization criteria: *d* > 0.2 as *small, d* > 0.6 as *moderate d* > 1.2 as *large*, and *d* > 2.0 as *very large* [30]. The level of significance was set at *p* < 0.05 and the statistical software used was SPSS for Windows (Version 26, IBM Corp., Armonk, NY, USA).

## RESULTS

**FIG. 1:**
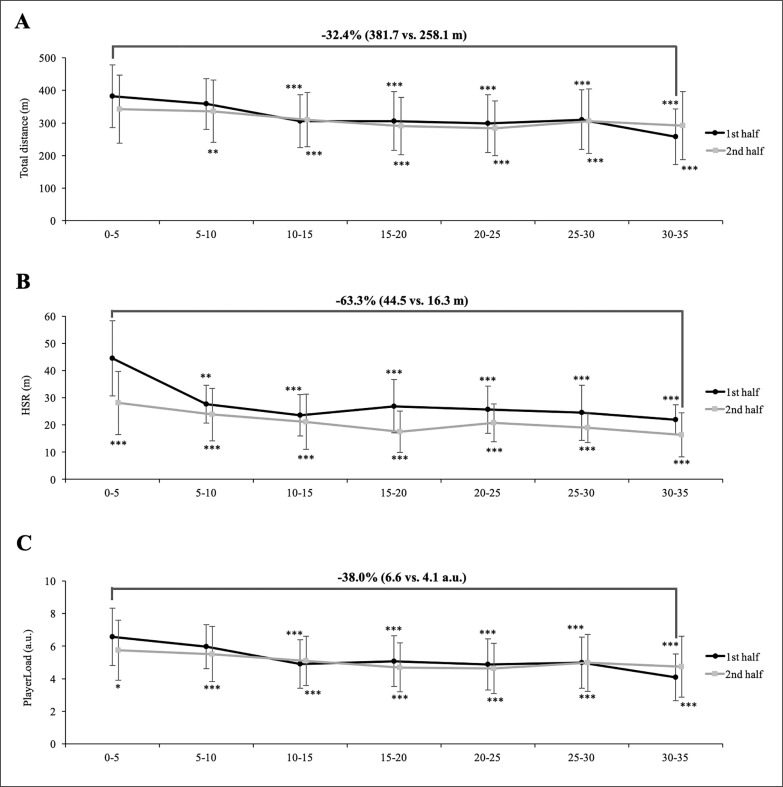
Differences according to match phases on external load experienced by handball players. Significant differences vs. first 5-min phase of the match = *p < 0.05; **p < 0.01: ***p < 0.001.

The physical performance fluctuated according to the match phase in all external load variables: TD (F = 10.30, p < 0.001, *η*p^2^ = 0.106), HSR (F = 9.13, p < 0.001, *η*p^2^ = 0.049) and PL (F = 11.22, p < 0.001, *η*p^2^ = 0.094) (Figure 1). More specifially, the match phase with the greatest mean physical performance was usually the first 5 min phase of the match (TD: 381.7 ± 95.9 m; HSR: 44.5 ± 37.8 m; PL: 6.6 ± 1.8 a.u.). However, the lowest mean physical performance was observed in the last 5 min phase of the first half (TD: 258.1 ± 85.6 m; PL: 4.1 ± 1.4 a.u.), except for HSR, which was registered in the last 5 min phase of the second half (16.3 ± 8.1 m). Additionally, players registered moderately lower values of TD (258.1 ± 85.6 m vs. 342.6 ± 104.2 m, p < 0.001, ES = 0.83) and PL (4.1 ± 1.4 a.u. vs. 5.8 ± 1.8 a.u., p < 0.001, ES = 0.98) during the last 5 min phase of the first half compared to the first 5 min phase of the second half.

**FIG. 2:**
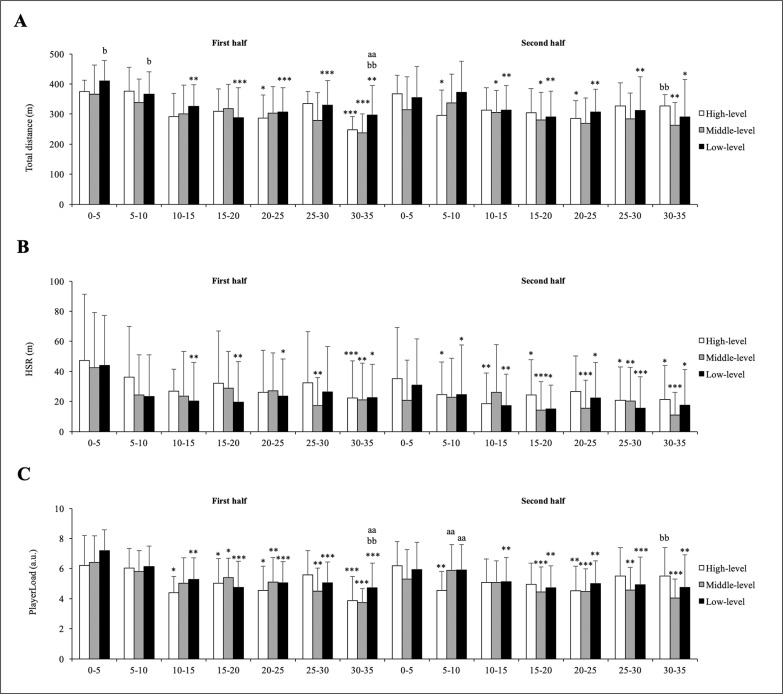
Differences according to level of the opponent and match phases on external load experienced by handball players. Significance level is indicated by the number of symbols: one symbol for p < 0.05, two for p < 0.01, and three for p < 0.001. a significant differences vs. high-level teams; b vs. middle-level teams; c vs. low-level teams. * significant differences vs. first 5-min phase of the match.

The level of the opponent had a significant effect on all external load variables: TD (F = 8.07, p < 0.001, *η*p2 = 0.012), HSR (F = 5.61, p = 0.004, *η*p2 = 0.009) and PL (F = 3.87, p < 0.021, *η*p2 = 0.006) (Figure 2). More specifically, matches involving LLT registered moderately more TD in the first (410.2 ± 68.1 m) and the second (365.8 ± 73.9 m) 5 min phases of the match compared to MLT (366.1 ± 96.5 m, p < 0.05, ES = 0.49; 338.5 ± 78.0 m, p < 0.05, ES = 0.51, respectively). Additionally, matches involving LLT registered moderately more TD (296.5 ± 98.0 m) and PL (4.7 ± 1.6 a.u.) in the last 5 min phase of the first half compared to HLT (247.0 ± 89.0 m, p = 0.009, ES = 0.74; 3.9 ± 1.6 a.u., p = 0.032, ES = 0.71, respectively) and MLT (237.9 ± 62.6 m, p = 0.008, ES = 0.82; 3.8 ± 0.9 a.u., p = 0.01, ES = 0.80, respectively). In contrast, matches involving HLT registered moderately more TD (326.6 ± 103.0 m) and PL (5.5 ± 1.9 a.u.) in the last 5 min phase of the second half compared to MLT (262.7 ± 75.4 m, p = 0.066, ES = 0.62; 4.0 ± 1.3 a.u., p = 0.011, ES = 0.81, respectively). However, no significant interactions between level of the opponent and match phases were observed for any variable.

**FIG. 3:**
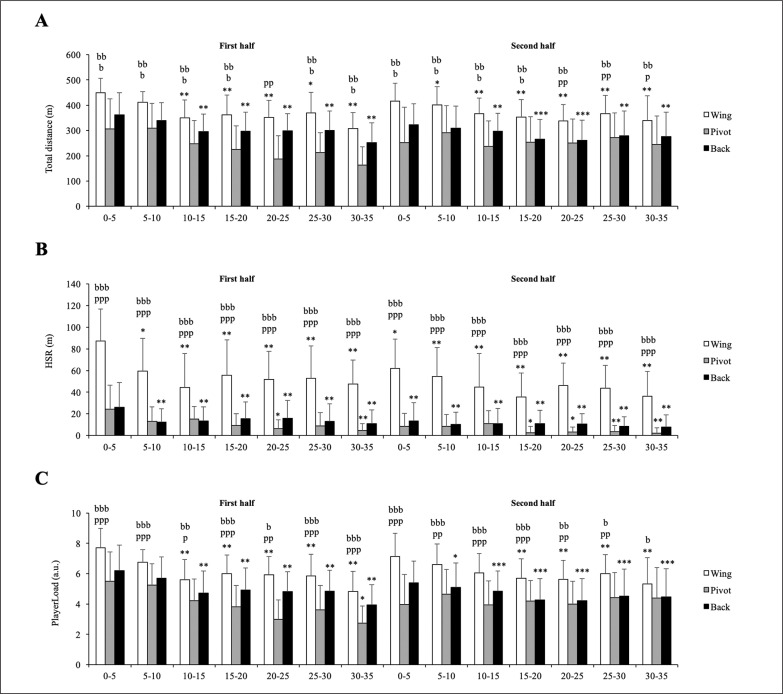
Differences according to playing positions and match phases on external load experienced by handball players. Significance level is indicated by the number of symbols: one symbol for p < 0.05, two for p < 0.01, and three for p < 0.001. b significant differences vs. backs; p vs. pivots; w vs. wings. * significant differences vs. first 5-min phase of the match.

The playing positions had a significant effect on all external load variables: TD (F = 153.29, p < 0.001, *η*p^2^ = 0.193), HSR (F = 539.53, p < 0.001, *η*p^2^ = 0.452) and PL (F = 122.44, p < 0.001, *η*p^2^ = 0.159) (Figure 3). Specifically, pivots showed the lowest physical performance in all 5 min phases of the match, whereas wings showed the highest physical performance in all variables. Additionally, pivots presented high variability between the most and the least intense 5 min phase while wings showed the lowest (Table 3). However, no significant interactions between playing positions and match phases were observed for any variable.

**TABLE 3:** Comparison between the most and the least intense 5-min fixed phase of the match for each playing position.

Variable	Playing positions	Most intense 5-min phase	Least intense 5-min phase	Difference	Difference (%)	*p*-value	Effect size (Cohen’s d)	

							Quantitative	Qualitative
Total distance (m)	Back	362.3 ± 86.6	251.4 ± 79.5	110.9 ± 7.1	30.6	p < 0.001	1.37	*Large*
	Pivot	308.9 ± 98.2	162.7 ± 72.2	146.2 ± 26.1	47.3	p = 0.098	1.32	*Large*
	Wing	449.1 ± 58.2	307.6 ± 63.9	141.5 ± 21.3	31.5	p < 0.001	1.95	*Very large*

HSR (m)	Back	26.1 ± 22.9	7.9 ± 11.1	18.2 ± 2.9	69.7	p < 0.001	1.23	*Large*
	Pivot	24.3 ± 22.3	2.1 ± 5.0	22.2 ± 5.4	91.2	p = 0.005	1.75	*Large*
	Wing	87.2 ± 29.5	36.3 ± 22.9	50.9 ± 7.5	58.5	p < 0.001	1.94	*Very large*

Player Load (a.u.)	Back	6.2 ± 1.7	4.0 ± 1.3	2.3 ± 0.4	36.4	p < 0.001	1.48	*Large*
	Pivot	5.5 ± 1.9	2.8 ± 1.1	2.7 ± 0.8	49.9	p = 0.021	1.54	*Large*
	Wing	7.7 ± 1.2	4.8 ± 1.3	2.9 ± 0.4	37.5	p < 0.001	2.09	*Very large*

## DISCUSSION

The aim of the study was to analyse physical performance fluctuations throughout match play in women’s handball and to investigate whether physical performance fluctuations are affected by contextual factors (i.e., level of the opponent and playing positions). The main findings associated with physical performance fluctuations indicated that: (1) the highest values of TD, HSR and PL were registered during the first 5 min phase of the match, regardless of the level of the opponent or playing position; (2) the lowest values of TD and PL were registered in the last 5 min phase of the first half, whereas in HSR they were registered in the last 5 min phase of the second half. Furthermore, the results connected to contextual factors were as follows: (1) there were significant differences with a small effect size between level of the opponent (HLT vs. MLT vs. LLT) for the first and the second 5 min phases of the first half in TD and for the last 5 min phase of each half in TD and PL; (2) there were significant differences with large effect sizes between playing positions (back vs. pivot vs. wing) for all 5 min phases in TD, HSR and PL.

In relation to physical performance fluctuations throughout the match, TD, HSR and PL were moderately higher during the first 5 min phase compared to the subsequent 5 min phases of the match, except for TD during the first 5 min phase of the second half where the values were similar to the first 5 min phase of the match. This information indicates that although the players started by testing the ball and shaking hands for approximately 30 seconds, the first 5 min phase of the first half were the most physically intense phase of the match. These results are consistent with previous findings from time-motion analysis [12, 16, 18, 19] and LPS analysis in hand-ball [14, 15]. There are many potential causes of these fluctuations in physical performance during the entire match. Firstly, at the beginning of the match, the teams try to dominate the opponents to obtain an advantage in the match score [15]. Secondly, the goal difference, especially if the match is already decided early in the second half, could affect the player’s pacing strategies and the coach’s decisions (e.g., experiment with new tactics or systems or introduce weaker or less-fit players) [8, 15]. Thirdly, the most likely reason, and one widely supported by scientific evidence, is that handball players experience fatigue during the match [14–20] and, consequently, their physical performance is reduced [31]. Our results suggest that handball players must be physically well prepared to tolerate an intense pace in the first stages of the match and to resist fatigue during the match. To accomplish this purpose, physical trainers and coaches should incorporate different interventions: (1) implement a suitable warm-up protocol before the match [32, 33]; (2) employ substitutions as an effective tool to distribute playing time between a larger number of players [8, 12, 15, 34]; (3) improve aerobic-anaerobic capacity and repeated-sprint ability of the players [15, 18].

In relation to the halves of the match, the first 5 min phase of each half registered moderately higher values of TD, HSR and PL compared to the subsequent 5 min phases of each half. Apparently, previous studies in handball reported similar results to our findings [15, 16, 19]. There are many factors that could explain these declines in physical performance within each half of the match: (1) repeat high-intensity actions (e.g., decelerations and changes of direction) associated with high eccentric contractions that generate important neuromuscular fatigue and tissue damage, especially if these high forces cannot be attenuated efficiently [35, 36]; (2) multiple collisions and contacts associated with one-on-one situations may produce tissue damage, inflammatory responses and neuromuscular impairments [18, 37]; (3) dehydration and hyperthermia, which affect cellular metabolism and accordingly performance [18]. Neverthless, the first 5 min phase of the second half registered moderately higher values of TD, HSR and PL compared to the last 5 min phase of the first half, but it was only significantly different for TD and PL. These data suggest that the beginning of the second half is a very physically demanding phase, although the HSR values are lower compared to the first 5 min phase of the match, probably because the teams do not play at a high pace to dominate the opponents. Consequently, it could be suggested that the half-time provides an opportunity to partially recover from the transient fatigue produced during the first half. Therefore, handball coaches and practitioners should consider the half-time break as a window of opportunity to include some strategies (e.g. rehydrate, re-fuel, ergogenic aids, heat maintenance, re-warm-up activities) to enhance or maintain physical performance at the beginning of the second half [33, 38]. Lastly, HSR values are lower in all 5 min phases of the second half compared to the first half, especially after the middle of the second half. This could be due to a slower pace of play and fewer counter-attacks as a specific strategy of the players to reduce the number of technical mistakes at the end of the match [12].

Positional differences were found in TD, HSR and PL, with wing players displaying the highest values and pivots showing the lowest values in all 5 min fixed phases. These results could be related to the technical and tactical demands of each playing position [4, 8, 14] and their position on the court [39], because the handball playing area is longer in the outer aisles than the central domain of the court because of the design of the goal areas, enabling wings to cover larger distances [39]. Furthermore, pivots presented a greater difference between the most and the least intense 5 min phase while wings showed a smaller difference. Several thoughts can be extracted from our findings: (1) this large variability between the most and the least intense 5 min phase reflects the great fluctuations in physical performance throughout the match in handball [15, 16, 19]; (2) the variability in physical performance is highly playing position-dependent [12]; (3) the technical staff should design and implement individualized physical training programmes for each playing position.

Regarding level of the opponent, matches involving LLT registered slightly more TD in the first and the second 5 min phase of the match compared to MLT, but it was not significantly greater compared to HLT. In contrast, matches involving HLT registered moderately more TD and PL in the last 5 min phase of the second half compared to MLT. We hypothesized that these results could be explained by two reasons: (1) LLT try to obtain an advantage in the match score at the beginning of the match; (2) matches involving HLT usually finished with a small goal difference; therefore, HLT players slowed the pace of the match in the middle of the second half to preserve energy for the last minutes of the match. Consequently, handball coaches should consider the level of the opponent to develop more effective game plans and player rotations strategies.

However, this study has some limitations. First, the external load was only monitored during home matches, which means that the match location could have influenced our results. Second, only data from 22 players were analyzed and all of them played in the same club. Third, an analysis of specialist players (offensive or defensive) and goalkeepers was not performed. Fourth, the playing position of each player was established exclusively according to her position in attack, without taking into account her defensive position. Fifth, as it was an observational study, player rotations could not be controlled or influenced by the researchers. Finally, physical performance during fixed phases was not as high as the worst case scenarios or most demanding passages calculated using the rolling average method [21, 22, 25, 28]. Hence, future research should investigate the worst case scenarios analyzing different women’s competitions and including a larger number of teams. In addition, future studies should include away matches.

## CONCLUSIONS

In the present study it was found that the physical performance fluctuated throughout the match and the fluctuations were affected by the level of the opponent and playing positions. Overall, the highest values of TD, HSR and PL were registered during the first 5 min phase of the match, while the lowest values of TD and PL were registered in the last phase of the first half and for HSR in the last phase of the second half. Regarding the level of the opponent, lowlevel teams elicited higher TD in the first 10 min of the match. In contrast, matches involving high-level teams registered more TD and PL in the last 5 min phase of the match. Additionally, in this investigation it was observed that playing positions had a significant effect on physical performance fluctuations. Wings showed the highest physical performance in all 5 min phases of the match, whereas the pivots showed the lowest physical performance. Consequently, physical trainers and handball coaches should incorporate different interventions to minimize the occurrence of fatigue within and between halves (e.g., player substitutions). Likewise, they should consider the half-time break as a window of opportunity to include some strategies (e.g. heat maintenance or re-warm-up) to enhance or maintain physical performance at the beginning of the second half.
